# Tumor Cell-Specific 2′-Fluoro RNA Aptamer Conjugated with *Closo*-Dodecaborate as A Potential Agent for Boron Neutron Capture Therapy

**DOI:** 10.3390/ijms22147326

**Published:** 2021-07-07

**Authors:** Mariya A. Vorobyeva, Maya A. Dymova, Darya S. Novopashina, Elena V. Kuligina, Valentina V. Timoshenko, Iaroslav A. Kolesnikov, Sergey Yu. Taskaev, Vladimir A. Richter, Alya G. Venyaminova

**Affiliations:** 1 Institute of Chemical Biology and Fundamental Medicine, Siberian Division of Russian Academy of Sciences, 630090 Novosibirsk, Russia; maya.a.rot@gmail.com (M.A.D.); danov@niboch.nsc.ru (D.S.N.); kuligina@niboch.nsc.ru (E.V.K.); timoshenkovalya@gmail.com (V.V.T.); katyono@mail.ru (I.A.K.); richter@niboch.nsc.ru (V.A.R.); ven@niboch.nsc.ru (A.G.V.); 2 Budker Institute of Nuclear Physics, Siberian Division of the Russian Academy of Sciences, 630090 Novosibirsk, Russia; taskaev@inp.nsk.su; 3 Department of Physics, Novosibirsk State University, 630090 Novosibirsk, Russia

**Keywords:** cell-specific aptamers, human glioblastoma cells, boron clusters, boron neutron capture therapy, cancer treatment, drug delivery

## Abstract

Boron neutron capture therapy (BNCT) is a binary radiotherapeutic approach to the treatment of malignant tumors, especially glioblastoma, the most frequent and incurable brain tumor. For successful BNCT, a boron-containing therapeutic agent should provide selective and effective accumulation of ^10^B isotope inside target cells, which are then destroyed after neutron irradiation. Nucleic acid aptamers look like very prospective candidates for carrying ^10^B to the tumor cells. This study represents the first example of using 2′-F-RNA aptamer GL44 specific to the human glioblastoma U-87 MG cells as a boron delivery agent for BNCT. The *closo*-dodecaborate residue was attached to the 5′-end of the aptamer, which was also labeled by the fluorophore at the 3′-end. The resulting bifunctional conjugate showed effective and specific internalization into U-87 MG cells and low toxicity. After incubation with the conjugate, the cells were irradiated by epithermal neutrons on the Budker Institute of Nuclear Physics neutron source. Evaluation of the cell proliferation by real-time cell monitoring and the clonogenic test revealed that boron-loaded aptamer decreased specifically the viability of U-87 MG cells to the extent comparable to that of ^10^B-boronophenylalanine taken as a control. Therefore, we have demonstrated a proof of principle of employing aptamers for targeted delivery of boron-10 isotope in BNCT. Considering their specificity, ease of synthesis, and large toolkit of chemical approaches for high boron-loading, aptamers provide a promising basis for engineering novel BNCT agents.

## 1. Introduction

Glioblastoma is the most frequent malignant brain tumor which remains incurable due to the rapid growth, invasive nature, and resistance to conventional therapies [1]. The particularity of its location complicates the development of glioblastoma therapies. The tumor localizes inside the brain, so the treatment must be precisely targeted because any side effects could bring dramatic consequences. Moreover, even targeted drugs should also come across the blood-brain barrier. Boron neutron capture therapy (BNCT) represents a promising approach to treating malignant tumors in general and glioblastoma in particular. BNCT is a binary radiotherapeutic modality based on the irradiation of stable boron-10 isotope by neutrons and subsequent production of high-energy alpha particles with very short pathlength (<10 µm) comparable to the diameter of one cell [2]. Therefore, they selectively destroy ^10^B-containing cells and spare ^10^B-free ones. Sufficient implementation of the BNCT concept in clinical medicine requires (1) selective accumulation of 1·10^9 10^B atoms per cell or 20 μg/g of tissue, and (2) proper neutron sources with optimal characteristics. To the moment, a number of neutron sources suitable for BNCT have been fabricated, including the neutron source developed in the Budker Institute of Nuclear Physics (BINP) (Novosibirsk, Russia) [2,3]. BNCT showed promising results in treating different tumors, including glioblastoma multiforme, meningioma, and head and neck cancers [4,5].

The only boron compounds clinically used for BNCT are boronophenylalanine (BPA) and sodium borocaptate (BSH), so-called second-generation boron delivery agents. In 2020, the Stella Pharma company marketed Steboronine^®^ drug (BPA as its D-sorbitol complex) for the treatment of head and neck cancer [6,7]. The pharmacokinetics and biodistribution of BSH and BPA are far from ideal, and one of their most prominent problems is the significant variability of tumor uptake [8]. Therefore, many research teams have focused on developing selective and effective boron delivery approaches to the tumor cells. Many low and high molecular weight third-generation boron delivery agents have been reported and showed promising characteristics in preliminary BNCT studies in vitro and in vivo (see [2,8,9] for recent comprehensive reviews in the field). Most of them consist of one or multiple boron clusters attached to the tumor-targeting molecule: Boron-loaded constructs can be addressed to tumor cells through specific peptides, antibodies, or other biomolecules affine to the cell surface proteins [2,9]. The use of dendrimers, liposomes, or nanotubes allows obtaining high boron-loaded agents [8,10].

Meanwhile, cell-specific DNA or RNA aptamers provide huge possibilities for tumor-addressed delivery. Aptamers bind tightly and specifically to their molecular targets, possess low toxicity and immunogenicity [11]. They are obtained by an automated chemical synthesis with minimal lot-to-lot variations and can be chemically modified to improve their biological stability [12]. A variety of aptamers and aptamer-based constructs have been reported, which can bind and internalize into the tumor cells, particularly glioma cells, in vitro and in vivo (see the reviews [13,14]). Moreover, different chemical approaches allow for multiple boron loading of synthetic nucleic acids [15]. Although the use of nucleic acid aptamers as possible BNCT agents was proposed as a concept nearly two decades ago [16], it has not yet been translated into practice. Here, we developed the cell-specific aptamer bearing terminal boron cluster as a potential BNCT agent for the first time and tested its effects in model BNCT studies after cell irradiation by epithermal neutrons. The use of 2′-fluoro-modified RNA aptamer loaded by *closo*-dodecaborate specifically decreased the viability of U-87 MG human glioblastoma cells to the extent comparable to that of BPA.

## 2. Results and Discussion

### 2.1. Candidate Aptamers: Testing Cellular Uptake and Cytotoxicity

For the successful implementation of the BNCT principle, boron atoms should be located inside the cell so that the cascade of reactions after neutron irradiation destroys basic cellular structures and processes, providing lethal effects. Accordingly, one of the main criteria for cell-specific aptamers as potential boron delivery vehicles for BNCT is their ability to bind and enter target cells. First, we analyzed the publications on nucleic aptamers specific to human glioblastoma cells U-87 MG and picked the aptamers reported to internalize inside the cells (see Table 1 for nucleotide sequences and references). We formed a series of 2′-F-RNA (nuclease-resistant RNA oligonucleotides with all pyrimidine nucleotides substituted by 2′-fluoro-modified analogs) and DNA aptamers with the Cy5 fluorophore (tetramethylindo(di)-carbocyanine 5) attached to the 3′-end via a flexible linker and tested their cellular uptake by confocal fluorescent microscopy. Scrambled 2′-F-RNA and DNA oligonucleotides (fscr and dscr, respectively, Table 1) labeled by Cy5, in the same manner, served as controls.

**Figure 1:**
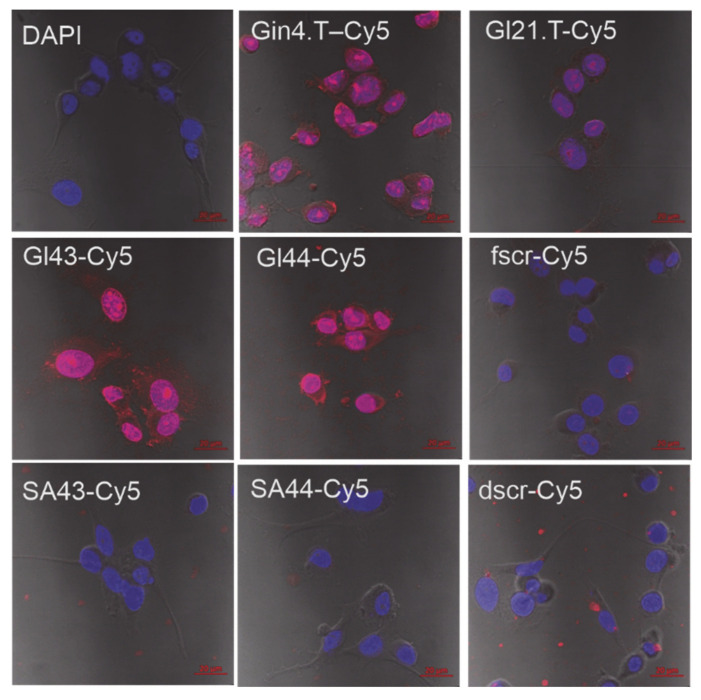
Cytochemical staining of human glioblastoma cells U-87 MG using Cy5-labeled 2’-F-RNA and DNA aptamers (see Table 1 for abbreviations and nucleotide sequences). DAPI-stained nuclei, blue signal, Cy5-labeled aptamers, and scrambled controls, red signal.

We carried out a comparative analysis of the efficiency of binding of the aptamers and control 2′-F-RNA and DNA oligonucleotides to the human glioblastoma cells U-87 MG by confocal microscopy with a confocal scanning microscope LSM 510 META (Zeiss) (Figure 1). According to the obtained images, the 2’-F-RNA aptamers Gin4.T, GL43, and GL44, readily internalized into the cells with the maximal intensity of Cy5 intracellular fluorescence for GL44, while GL21.T aptamer provided lower penetration efficiency. All 2′-F-RNA aptamers penetrated the cell nucleus; the images show a clearly stained nucleus with nucleoli, the cytoplasm was also stained. At the same time, scrambled 2′-F-RNA control fscr showed inferior cell penetration.

In contrast, we registered no specific cell internalization for both DNA aptamers SA43 and SA44, as well as for corresponding control DNA (dscr) (Figure 1). These results are quite controversial with the data obtained for these aptamers by Aptekar et al. [19] on the same model cell line. The only noticeable difference in our assay was the use of Cy5 fluorescent dye instead of the Cy3 fluorophore employed in [19]. However, this replacement can hardly cause such an effect, so the question remains open.

During the incubation of U-87 MG cells with 2’-F-RNA aptamer Gin4.T, we observed some changes in the morphological structure of cells. The cells lost their fusiform shape, lost processes, and acquired a rounded shape, which may indirectly indicate the cytotoxicity of the aptamer at a given concentration (2.5 μM). It is important to note that these results are consistent with the data on the cytotoxicity of these aptamers to tumor cells, described in the original works on their selection and properties [17,21].

Since 2’-F-RNA aptamer GL44 showed very good cell penetration and nuclear localization, we chose this particular aptamer as a candidate boron delivery agent for model BNCT experiments.

### 2.2. Synthesis of Terminal Conjugates of 2′-F-RNAs with Closo-Dodecaborate

In the present study, we covalently attached the boron cluster to the 5′-end of oligonucleotides using the method developed by us earlier [22,23] for oligonucleotides of different types, including 2′-F-RNA. It is important to note that *closo*-dodecaborate residues located at the terminal positions of 2′-F-RNA do not change the structure of their homoduplexes and their stability [22]. Therefore, we proposed that analogous terminal modification should not disturb the structure of duplex fragments within the 2′-F-RNA aptamer, so it will most likely retain target binding affinity.

**Figure 2:**
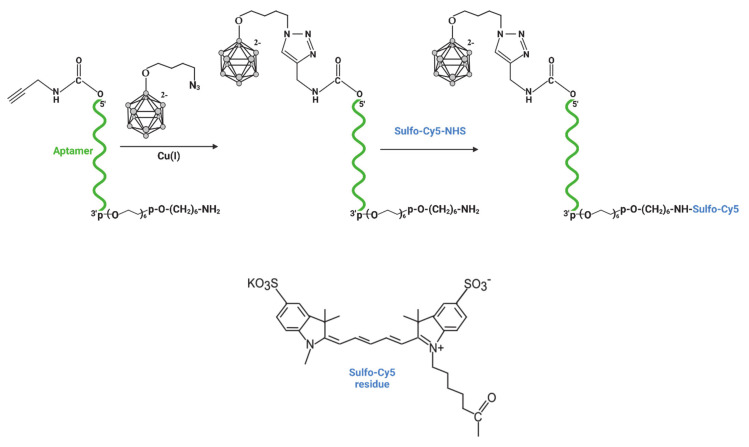
Scheme of the synthesis of bifunctional 2’-F-RNA conjugates bearing 5′-terminal *closo*-dodecaborate residue and 3′-terminal sulfo-Cy5 fluorescent dye.

The aptamer GL44 and scrambled control 2′-F-RNA fscr (Table 1) bearing boron cluster at 5’-end and fluorophore at 3’-end were obtained step by step starting from 3’-amino-modified oligonucleotides immobilized at the solid support (see Figure 2 for the reaction scheme). First, the alkyne group was attached to the oligonucleotide using the 5’-hydroxyl activation by N,N′-disuccinimidyl carbonate (DSC) [22] and subsequent interaction with propargylamine. Then, after deprotection and release of 5’-alkyne-3’-amino-modified oligonucleotides from CPG-support, *closo*-dodecaborate azide (*closo*B_12_-azide) was attached to 5’-alkyne via Cu-catalyzed click chemistry. Next, the introduction of fluorophore at 3’-end was carried out by the interaction of the 3’-amino group with the NHS ester of Sulfo-Cyanine 5 (Sulfo-Cy5). Finally, both conjugates were isolated by denaturing gel electrophoresis analysis and characterized by mass spectrometry (Table 2).

### 2.3. Cell Penetration and Cytotoxicity of Aptamer-Boron Cluster Conjugates

**Figure 3:**
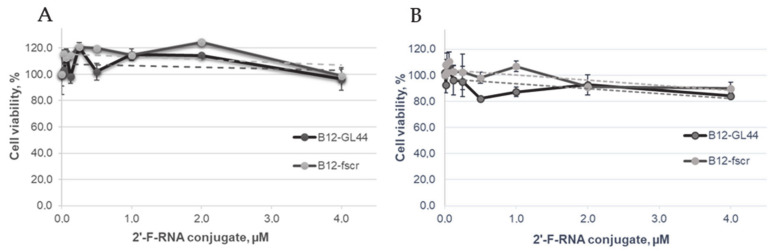
The results of the MTT assay represented as cell viability curves after treatment by the *closo*-dodecaborate-modified 2′-F-RNA conjugates B_12_-GL44 or B_12_-fscr. (**A**)—U-87 MG glioblastoma cells, (**B**)—normal human fibroblasts hFF8.

The effects of bifunctional *closo*-dodecaborate-modified 2’-F-RNAs B_12_-GL44 and B_12_-fscr on the viability of U-87 MG human glioblastoma cells and normal hFF8 fibroblast cells was determined by the standard colorimetric 3-(4,5-dimethyl-2-thiazolyl)-2,5-diphenyl-2H-tetrazolium bromide (MTT) assay. We assessed their cytotoxicity in the concentration range of 15.6 nM to 4 µM; cell viability curves are given in Figure 3.

The cytotoxicity analysis revealed the non-toxic nature of the *closo*-dodecaborate 2′-F-RNA conjugates B_12_-GL44 or B_12_-fscr. Both of them did not trigger significant loss of U-87 MG and hFF8 cells viability even at high concentrations. Therefore, we could safely use the conjugates at micromolar concentrations in model BNCT experiments.

**Figure 4:**
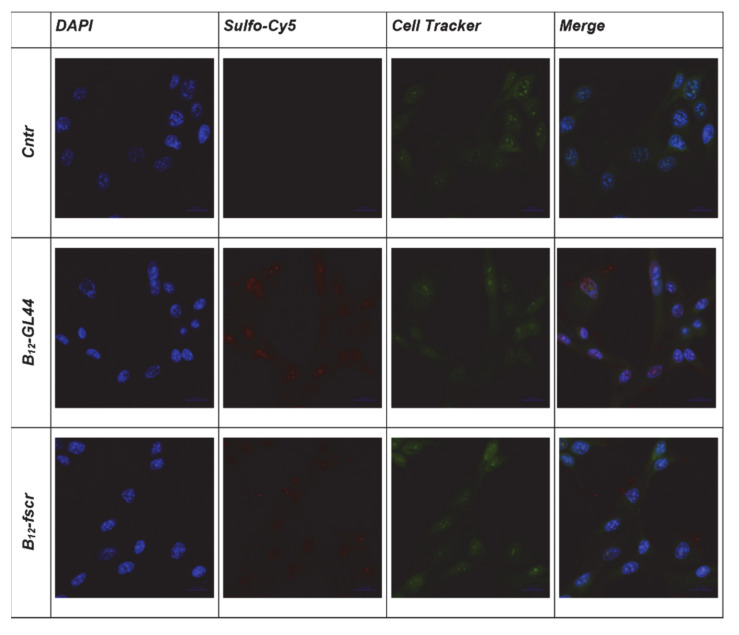
Cytochemical staining of human glioblastoma cells U-87 MG using the *closo*-dodecaborate conjugates of 2’-F-RNA aptamer B_12_-GL44 or scrambled control B_12_-fscr. DAPI-stained nuclei, blue signal, Sulfo-Cy5-labeled 2’-F-RNA conjugates, red signal, CellTracker ™ green CMFDA, green signal.

Before BNCT experiments, we also evaluated the uptake of the B_12_-GL44 2′-F-RNA aptamer conjugate by U-87 MG glioma cells. We carried out a comparative analysis of the efficiency of binding of B_12_-GL44 and control B_12_-fscr conjugate to the human glioblastoma cells U-87 MG and normal fibroblast cells hFF8 by confocal microscopy. The operating value of the exposure for the Cy5 channel was the same for all analyzed samples. Staining U-87 MG cells using bifunctional 2’-F-RNA conjugates (Figure 4) showed that the boron-containing aptamer conjugate B_12_-GL44 interacts more efficiently with cells than scrambled control B_12_-fscr, which demonstrated a low internalization level. The images show a clearly stained nucleus with nucleoli, so we concluded that the B_12_-GL44 conjugate penetrates the cell nucleus.

**Figure 5:**
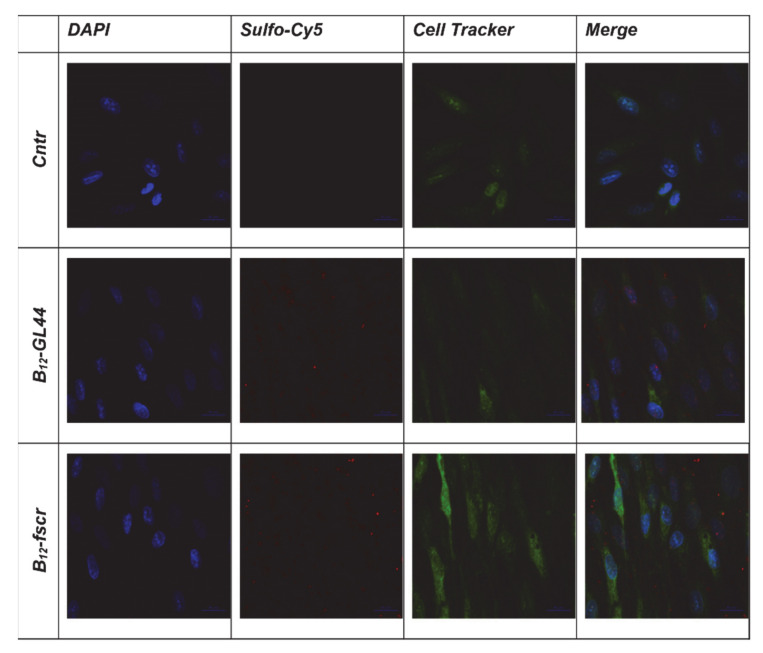
Cytochemical staining of normal fibroblast cells hFF8 using the conjugates of 2’-F-RNA aptamer GL44 or scrambled 2′-F-RNA fscr with *closo*-dodecaborate. DAPI-stained nuclei, blue signal, Sulfo-Cy5-labeled 2’-F-RNA conjugates, red signal, CellTracker ™ green CMFDA, green signal.

In the case of normal hFF8 fibroblast cells, both B_12_-GL44 and B_12_-fscr conjugates bound non-specifically to the cell surface in small quantities (Figure 5) without cell internalization. Therefore, we assumed that in model BNCT experiments, they would not have significant effects on normal fibroblast cells.

### 2.4. The Effect of Aptamer-Boron Cluster Conjugate on Cell Viability after Irradiation

#### 2.4.1. Cell Incubation and Treatment before Irradiation

**Figure 6:**
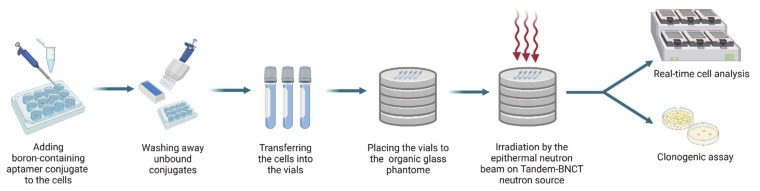
The schematic representation of experimental protocol for in vitro BNCT and subsequent assessment of cell viability.

In general, the protocol of model in vitro BNCT experiments included the following steps: (1) incubation of the cells with boron-containing 2′-F-RNA; (2) washing away the non-internalized conjugates; (3) neutron irradiation of the cells; (4) assessment of the cell viability after irradiation (Figure 6).

We chose the concentrations of aptamer-*closo*-dodecaborate for model BNCT experiments considering the number of cells in each sample and the demand that at least 10^9 10^B atoms should be accumulated in each glioma cell. As the *closo*-dodecaborate used in our study consists of natural boron with approx. 20% of boron-10 isotope_,_ this information was also taken into account. The final concentration of boron-containing conjugates was 1.7 μM. As shown in the previous section, this concentration should not cause any cytotoxic effects for U-87 MG and hFF8 cells.

Before the irradiation on the Tandem-BNCT neutron source, the cells were incubated with the conjugate B_12_-GL44 or B_12_-fscr, then washed, resuspended in complete culture media, and transferred to the 2 mL vials. To evaluate the specificity of the effects brought by aptamer treatment and subsequent neutron irradiation, we formed a series of control samples: (1) U-87 MG glioblastoma cells and normal hFF8 fibroblast cells treated by B_12_-GL44 or B_12_-fscr without irradiation; (2) the same cells irradiated without pre-treatment by the conjugates; and (3) cells free from both aptamer treatment and irradiation.

#### 2.4.2. Neutron Irradiation Using the BINP Neutron Source

Irradiation of cell cultures was carried out at the BINP neutron source [3]. Regarding the choice of radiation dose, we relied on previously obtained data [24] showing that irradiation with these particular accelerator settings and relevant concentrations produces a therapeutic effect.

#### 2.4.3. The xCELLigence Real-Time Cell Analysis (RTCA)

To assess the viability of U-87 MG cells and control fibroblasts hFF8, we monitored the cell proliferation using an iCELLigence real-time cell analysis system [25]. All cells were seeded in two 8-well microtiter plates at a density of 30,000 cells per well in double after irradiation.

**Figure 7:**
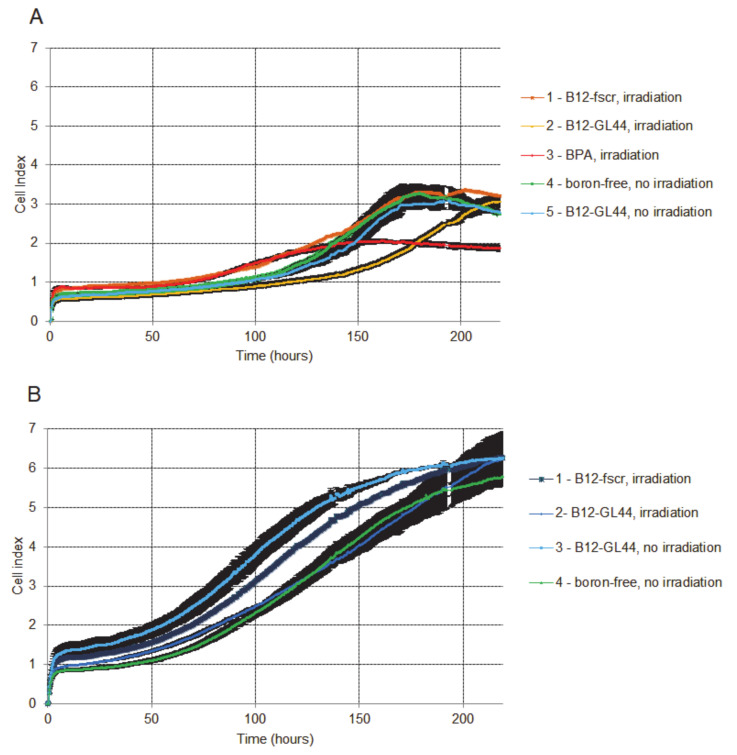
Dynamic RTCA monitoring of cell adhesion and proliferation of human glioblastoma cells U-87 MG (**A**) and normal fibroblast cells hFF8 (**B**) after incubation with boron-containing 2′-F-RNAs or BPA and irradiation with epithermal neutrons.

The proliferation curve for U-87 MG cells irradiated after the treatment by the boron-containing 2′-F-RNA aptamer conjugate B_12_-GL44 (Figure 7A, curve 2) shows the lowest cell index (CI) in all U-87 MG series up to 175 h of monitoring. After that, the cell index began to rise and reached the level of non-irradiated control cells (Figure 7A, curve 4). It is important to note that non-irradiated cells treated by B_12_-GL44 demonstrated nearly the same CI curve (Figure 7A, curve 5) as control non-treated cells without irradiation, proving that both aptamer treatment and neutron irradiation are necessary to suppress the cell viability.

In contrast, the cells irradiated in the presence of BPA showed higher CI values at the beginning of monitoring (Figure 7A, curve 3) but a more prolonged inhibiting effect with the lowest cell index at the end of monitoring. After 175 h of monitoring, this group of cells demonstrated lower CI than cells treated by a boron-containing aptamer. Of note, the control scrambled 2′-F-RNA conjugate B_12_-fscr provided nearly the same CI curve as for control non-irradiated conjugate-free U-87 MG cells (Figure 7A, curves 1 and 4). This evidences the specific inhibition of cell viability by the aptamer-driven boron cluster.

In the case of control human fibroblasts hFF8 (Figure 7B), the cells irradiated after B_12_-GL44 (Figure 7B, curve 2) treatment showed the CI curve close to that for control aptamer-free cells without irradiation (Figure 7B, curve 4). At the same time, B_12_-fscr treatment and irradiation gave nearly the same proliferation curve (Figure 7B, curve 1) as B_12_-GL44 treated non-irradiated fibroblasts (Figure 7B, curve 3). We interpreted these results as the absence of any pronounced effects of model BNCT for control non-tumor cells.

Therefore, we demonstrated for the first time the specific inhibition of tumor cell proliferation in model BNCT experiments with 2′-F-RNA aptamer as boron delivery agent.

#### 2.4.4. Clonogenic Assay

We also made an independent assessment of cell survival after irradiation by the ability of the cells to form colonies using the clonogenic assay. This assay is widely used in model BNCT studies, e.g., to optimize the irradiation parameters [26] or characterize novel of different boron delivery agents [27].

**Figure 8:**
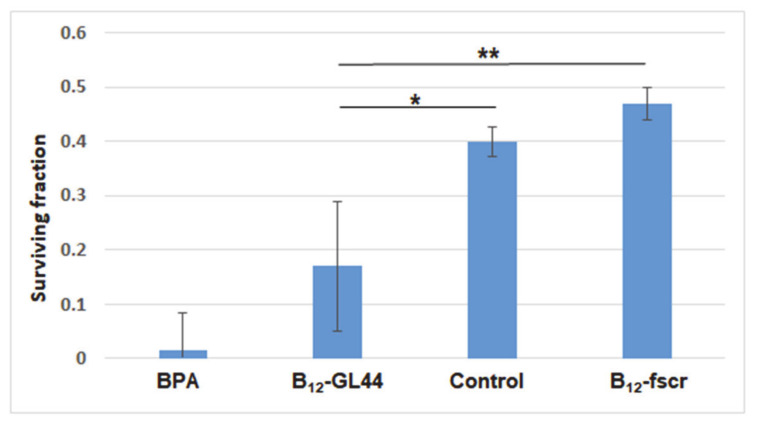
Surviving fractions of neutron-irradiated human glioblastoma cells U-87 MG depending on the incubation with the boron-containing 2′-F-RNA aptamer conjugate B_12_-GL44, scrambled 2′-F-RNA conjugate B_12_-fscr or BPA. Control cells were irradiated without pre-treatment by any boron compounds. * p ≤ 0.05; ** p ≤ 0.01.

The results of clonogenic assay for U-87 MG cells were normalized to the survival rate of untreated non-irradiated cells (see the diagram in Figure 8). After neutron irradiation, the survived fractions of U-87 MG cells treated by BPA or aptamer conjugate B_12_-GL44 were 0.015 and 0.17, respectively. In contrast, boron-containing scrambled 2′-F-RNA gave a 0.47 survival rate after irradiation, close to that for control conjugate-free irradiated cells (0.40). There were no statistical differences between survived fractions of B_12_-Gl44 and BPA-treated cells and between B_12_-fscr treated cells and control ones. Meanwhile, we found statistical differences between survived fractions of the cells treated by B_12_-Gl44 and B_12_-fscr (*p* ≤ 0.01) and between B_12_-Gl44 treated cells and control cells (*p* ≤ 0.05). This data shows that physiologically relevant concentrations of boron-loaded 2’-F-RNA aptamers can decrease the cell viability in model BNCT experiments.

In the case of control non-tumor cells, normal fibroblasts hFF8, we failed to perform the clonogenic assay. These cells formed very diffuse colonies on Petri dishes, making it impossible to quantify the results. However, the cells were viable, divided, and did not lose their morphology, which partly proves that they mostly retained their viability after treatment and irradiation.

The clonogenic assay results nicely coincide with the data from cell viability monitoring described in the previous section (Section 2.4.3). Both assays clearly demonstrate the specific decrease of cell viability after neutron irradiation of the tumor cells treated by boron-containing aptamer conjugate, comparable to that for BPA. However, the long-term effect of the BPA is more prominent as yet, as judged from the end-point CI values from RTCA (after 218 h) and cell viabilities from the clonogenic assay. We hypothesize that the incomplete inhibition of tumor cell viability could be explained by the presence of glioma stem/progenitor cells (GSPC) fraction, more resistant to ionizing radiations [28]. This posts a task of additional optimizing the concentration of the aptamer conjugate and the mode of neutron irradiation in further studies. Nevertheless, repeated doses of conjugate treatment and irradiation will likely provide a sustainable decrease of tumor cell viability.

We would also like to emphasize that *closo*-dodecaborate used in this work contains natural boron with only 20% of ^10^B isotope. However, even in this case, we observed the pronounced biological effect. There is a strong probability that the use of boron-10 enriched clusters of the increase of the number of clusters per one aptamer would provide more prominent inhibition of cell viability.

## 3. Materials and Methods

### 3.1. Chemicals and Reagents

3’-PT-Amino-Modifier C6 CPG, 5′,N-protected 2′-O-TBDMS-ribo- (A and G), 5′,N-protected 2’-deoxyribophosphoramidites, Spacer Phosphoramidite 18, and 3’-PT-Amino-Modifier C6 polymer support were purchased from Glen Research Inc (Sterling, VA, USA). 5′,N-Protected 2’-deoxy-2’-fluoro pyrimidine phosphoramidites were purchased from ChemGene Corp (Wilmington, MA, USA). N,N-Diisopropylethylamine (DIPEA), and propargylamine were purchased from Sigma-Aldrich (St. Louis, MO, USA), N,N′-disuccinimidyl carbonate (DSC) was purchased from Acros Organics (Geel, Belgium), Sulfo-Cyanine 5 NHS ester, 10 mM Cu(II)-Tris(benzyltriazolylmethyl)amine (Cu(II)-TBTA) stock in 55% dimethyl sulfoxide (DMSO), ascorbic acid were purchased from Lumiprobe (Russia), and sodium dodecaborate Na_2_[B_12_H_12_] was from AviaBor (Dzerzhinsk, Russia). ^10^B-enriched (>99%) BPA) was purchased from Katchem spol. s r. o. (Czech Republic), and converted into fructose 1:1 complex for increasing solubility [29]. All solvents (tetrahydrofuran, DMSO, CH_3_CN (various vendors)) were dried by 3 Å molecular sieves or by distillation and stored over CaH_2_. Bis-tetrabutylammonium-(4-azidobuthoxy)-undecahydro-*closo*-dodecaborate (*closo*B12-azide) was synthesized as described in [22] and kindly provided by Dr. V.N. Silnikov (ICBFM SB RAS).

### 3.2. Cell Lines

The human glioblastoma cell line U-87 MG was obtained from the Russian cell culture collection (Russian Branch of the ETCS, St. Petersburg, Russia). The normal human fibroblasts hFF8 were kindly provided by Dr. Filipenko M.L. (Laboratory of Pharmacogenomics, ICBFM SB RAS). U-87 MG and hFF8 cell lines were cultured in Minimum Essential Media (MEM) and Iscove’s Modified Dulbecco’s Medium (IMDM) (Gibco, Waltham, MA, USA), respectively, supplemented with 10% (*v*/*v*) of fetal bovine serum (Gibco, Waltham, MA, USA) and 1% (*v*/*v*) antibiotic-antimycotic solution (Gibco, Waltham, MA, USA). Cells were maintained at 37 °C in a 5% CO_2_ atmosphere.

### 3.3. Synthesis of Oligonucleotides

2′-F-RNA and DNA aptamers and control scrambled oligonucleotides (fscr, AC^F^U^F^GGU^F^AU^F^GU^F^C^F^GAGC^F^C^F^AAC^F^AAU^F^C^F^GAU^F^AC^F^C^F^AAGAC^F^U^F^AAGA; dscr ATACGTTAACGATCCTTCACTACACCTATAATATCCTGTTGAT) were synthesized by the solid phase phosphoramidite method on 0.4 µmol scale on an automated DNA/RNA synthesizer ASM-800 (Biosset, Novosibirsk, Russia) using corresponding 5′,N-protected phosphoramidites of 2’-O-tert-butyldimethylsilyl (2’-O-TBDMS) ribonucleotides, 2’-fluoro-2’-deoxyribonucleotides or deoxyribonucleotides and protocols optimized for the instrument. Oligonucleotides bearing a 3’-amine group were synthesized using modified polymer support 3’-PT-Amino-Modifier C6 CPG and Spacer Phosphoramidite 18. Oligonucleotides with 5’-alkyne modification were obtained in analogy with [16]. After synthesis, cleavage from support and deprotection of the oligodeoxyribonucleotides were carried out with 300 μL of 40% aq. methylamine solution at 65 °C for 15 min. 2′-F-RNA oligonucleotides were deprotected by 300 μL of AMA solution (ammonium hydroxide/40% aq. methylamine 1:1 *v*/*v*) at 25 °C for 2 h. The 2′-O-TBDMS groups of purine ribonucleotides were removed using 200 μL of mixture NMP/TEA·3HF/TEA (150/100/75) at 65 °C for 1.5 h, followed by the treatment by 300 μL of trimethylethoxysilane (TCI, Portland, OR, USA) and precipitation with diethyl ether.

### 3.4. Synthesis of Cy5-Labeled Aptamers

3’-Amino modified oligonucleotides (25 nmol) were dissolved in 20 μL of 0.05 M Tris-HCl buffer (pH 7.8) and 1 mg (1.5 µmol) of Cyanine 5 NHS ester dissolved in 80 μL DMSO was added. The reaction mixture was incubated at room temperature for 2 h. Oligonucleotide conjugates were precipitated with 2% NaClO_4_ in acetone and washed with acetone. The pellets were dried on-air, dissolved in water, and purified using Amicone 3K microcentrifuge modules (Millipore, Billerica, MA, USA).

### 3.5. Synthesis of Bifunctional 2’-F RNA Conjugates with Closo-Dodecaborate and Sulfo-Cy5

Triethylammonium acetate buffer (pH 7.0), 10 mM *closo*B_12_-azide in DMSO, 5 mM ascorbic acid solution in water, and 10 mM Cu(II)-TBTA stock in 55% DMSO were added to the water solution of 5′-alkyne-modified 2′-F-RNA (25 nmol) according to the protocol of click reagent supplier (Lumiprobe, Moscow, Russia). The reaction mixture was incubated at room temperature overnight. Oligonucleotide conjugates were precipitated with 2% NaClO_4_ in acetone and washed with acetone. The pellets were dried on-air, dissolved in water, analyzed, and isolated by gel electrophoresis.

The resulting 3’-amino-modified 2’-F-RNAs bearing 5’-*closo*-dodecaborate (25 nmol) were dissolved in 20 μL of 0.05 M Tris-HCl buffer (pH 7.8) and 0.4 mg (0.5 µmol) of Sulfo-Cyanine 5 NHS ester dissolved in 80 μL DMSO was added. The reaction mixture was incubated at room temperature for 2 h. Oligonucleotide conjugates were precipitated with 2% NaClO_4_ in acetone and washed with acetone. The pellets were dried on-air, dissolved in water, analyzed, and isolated by gel electrophoresis. The purified oligonucleotide conjugates were characterized by ESI mass-spectrometry using ESI LC/MS/MSD XCT instrument (Agilent Technologies, Santa Clara, CA, USA) (Table 2).

### 3.6. Confocal Microscopy

Fluorescent staining of cells was carried out according to the technique described in [19] with minor modifications. U-87 MG cells were incubated on BD Falcon culture slides to 80–90% confluence, washed with phosphate buffered saline (PBS) twice. The solution of fluorescently labeled DNA or 2′-F-RNA aptamer (200 nM in αMEM) was heated to 95 °C for 5 min, then ice-cooled for 2 min and incubated at 37 °C for 15 min. The cells were incubated with aptamers for 30 min at 37 °C, in the dark, at 50 rpm, and washed with PBS (5 × 250 μL). Then 200 μL/well of cold methanol were added and incubated for 10 min at 4 °C. Next, the cells were washed with cold PBS (2 × 250 μL), supplied with 2.5 μM CellTracker™ green CMFDA dye, and incubated at 37 °C in the dark, then washed twice with PBS. After that, the cells were stained with 4′,6-Diamidino-2-phenylindole (DAPI) (Thermo Fisher Scientific, Waltham, MA, USA USA) and analyzed by fluorescent microscopy Axio Skope 2 Plus (Zeiss, Oberkochen, Germany) at the Center for Microscopic Analysis of Biological Objects of SB RAS (Novosibirsk, Russia). The background level of cell autofluorescence was determined using preparations of unstained controls. The operating value of the exposure for the Cy5 channel was the same for all analyzed samples and amounted to 760 ms.

### 3.7. Cytotoxicity Analysis

Cytotoxicity analysis was performed using an MTT assay. Two cell lines were seeded in 96-well plates at a density of 5·10^3^ cells per well. After 24 h of incubation, they were incubated with different concentrations of the conjugates, from 4 µM to 15.6 nM using double serial dilutions for another 24 h. After that, cell proliferation was assessed using a 3-(4,5-dimethylthiazol-2-yl)-2,5-diphenyl tetrazolium bromide (MTT) assay. Optical density was recorded using a microplate reader at 570 nm, with a reference wavelength of 620 nm. The following analysis and dose-effect curves were performed using CompuSyn software [30,31].

### 3.8. Model BNCT Experiments

Human glioblastoma U-87 MG cells (0.5·10^6^ cells per well) were incubated for 30 min with B_12_-GL44 or B_12_-fscr 2′-F-RNA conjugates (800 μL of 1.7 μM in αMEM) for 30 min, then washed by 1 mL of complete culture medium. To obtain a positive BCNT control, U-87 MG cells were incubated with ^10^B-4-borono-L- phenylalanine (20 µg ^10^B/mL) for 18 h, at 37 °C in a 5% CO_2_ atmosphere.

Control human fibroblasts were treated with B_12_-GL44 or B_12_-fscr 2′-F-RNA conjugates as described above for U-87 MG cells.

The cells without aptamer conjugates and ^10^B-BPA were irradiated and used as controls.

Before irradiation, all samples were transferred into 2 mL vials and placed to the phantom made of organic glass. Then, neutron irradiation was performed at the vacuum-insulation tandem accelerator at BINP SB RAS for 1 h under the following conditions: 2 MeV proton energy, 1.4 mA proton current, epithermal neutron fluence 5·10^11^ neutrons/cm^2^.

### 3.9. The xCELLigence Real-Time Cell Analysis (RTCA)

After irradiation, U-87 MG cells or control hFF8 fibroblasts cells were seeded in two 8-well microtiter plates at a density of 3·10^4^ cells per well. The impedance value of each well was automatically monitored in real-time by the xCELLigence RTCA system (Technologies, Santa Clara, CA, USA) for a duration of 218 h and expressed as a CI (cell index) value [25].

### 3.10. Clonogenic Assay

After irradiation, the cells were seeded in 6-well plates (TPP, Trasadingen, Switzerland) at a density of 300 cells per well and incubated at 37 °C in a humidified incubator under 5% (*v*/*v*) CO_2_ for a week. Colonies were fixed with glutaraldehyde (6.0% *v*/*v*) and stained with crystal violet (0.5% *w*/*v*) [32]. Colonies of more than 50 cells were counted. The percent plating efficiency and surviving fraction were calculated based on the survival of non-irradiated cells.

### 3.11. Statistical Analyses

Outcome variables are expressed as means ± standard deviations (SDs). Each experiment was repeated at least three times. Statistical analysis was performed using GraphPad Prism 6.01 (GraphPad Software, San Diego, CA, USA). Two-way ANOVA was used for comparisons of more than two sets of data. Differences were considered to be significant if the *p*-value was <0.05.

## 4. Conclusions

To sum up, this study represents the very first example of employing nucleic acid aptamer as a tumor cell-specific boron carrier for boron neutron capture therapy. We formed a series of previously published 2′-F-RNA and DNA aptamers, which were reported to internalize into U-87 MG human glioblastoma cells specifically. We compared their internalization by confocal microscopy and chose 2′-F-RNA aptamer GL44 which effectively penetrates the cell nuclei. This aptamer was then supplied with *closo*-dodecaborate residue at the 5′-end and fluorophore at the 3′-end. The resulting bifunctional conjugate showed specific tumor cell internalization together with low toxicity in the MTT assay. We then tested the boron-containing conjugate as a boron delivery agent in model BNCT experiments in vitro. Their results were evaluated by two independent methods, which revealed that *closo*-dodecaborate conjugate of 2′-F-RNA aptamer GL44 provides the specific decrease of tumor cells viability after neutron irradiation, comparable to that of ^10^B-BPA, the ‘gold standard’ of BNCT. In this pilot study, the aptamer conjugate had a lesser long-term effect than BPA. We suggest that further optimization of the conjugate concentration, dosage, and irradiation mode would bring even more prominent biological effects. Meanwhile, in this study, we employed the boron cluster made of natural boron-containing only 20% of ^10^B. Using boron-10 enriched clusters or attaching several clusters to the aptamer molecule would give us very promising aptamer-based BNCT agents.

## Figures and Tables

**Table 1:** Candidate aptamers with reported ability to internalize U-87 MG cells and control scrambled oligonucleotides.

Aptamer	Nucleotide Sequence, 5′→3′	Ref.
Gin4.T	U^F^GU^F^CGU^F^GGGGC^F^AU^F^C^F^GAGU^F^AAAU^F^GC^F^AAU^F^U^F^C^F^GAC^F^A	[17]
GL21.T	AU^F^GAU^F^C^F^AAU^F^CGCCU^F^C^F^AAU^F^U^F^C^F^GAC^F^AGGAGGC^F^U^F^C^F^AC^F^	[18]
GL43	AC^F^GU^F^U^F^AC^F^U^F^C^F^U^F^U^F^GC^F^AAC^F^AC^F^AAAC^F^U^F^U^F^U^F^AAU^F^AGC^F^C^F^U^F^C^F^U^F^U^F^AU^F^AGU^F^U^F^C^F^	[19,20]
GL44	AC^F^GU^F^U^F^AC^F^U^F^C^F^U^F^U^F^GC^F^AAC^F^AC^F^C^F^C^F^AAAC^F^U^F^U^F^U^F^AAU^F^AGC^F^C^F^U^F^C^F^U^F^U^F^AU^F^AGU^F^U^F^C^F^	[19,20]
SA43	ACGTTACTCTTGCAACACAAACTTTAATAGCCTCTTATAGTTC	[19]
SA44	ACGTTACTCTTGCAACACCCAAACTTTAATAGCCTCTTATAGTTC	[19]
fscr	AC^F^U^F^GGU^F^AU^F^GU^F^C^F^GAGC^F^C^F^AAC^F^AAU^F^C^F^GAU^F^AC^F^C^F^AAGAC^F^U^F^AAGA	
dscr	ATACGTTAACGATCCTTCACTACACCTATAATATCCTGTTGAT	

Here and after, C^F^—2’-deoxy-2′-fluorocytidine, U^F^—2’-deoxy-2′-fluorouridine.

**Table 2:** Bifunctional conjugates of 2’-F RNA aptamer and scrambled control with *closo*-dodecaborate and sulfo-Cy5 residues.

Conjugate	Sequence, 5’-3’	Molecular Weight, Da	
		Calc.	Found
B_12_-GL44	5’-*closo*B_12_-AC^F^GU^F^U^F^AC^F^U^F^C^F^U^F^U^F^GC^F^AAC^F^AC^F^C^F^-C^F^AAAC^F^U^F^U^F^U^F^AAU^F^AGC^F^C^F^C^F^U^F^U^F^AU^F^AGU^F-^U^F^C^F^p-L-NH-Sulfo-Cy5	15,777.4	15,781.7
B_12_-fscr	5’-*closo*B_12_-AC^F^U^F^GGU^F^AU^F^GU^F^C^F^GAGC^F^C^F^AAC^F^A-AU^F^C^F^GA-U^F^AC^F^C^F^AAGAC^F^U^F^AAGA-p-L-NH-Sulfo-Cy5	14,041.6	14,047.5

*closo*B_12_**_—_***closo*-dodecaborate residue; L—flexible spacer **-**(OCH_2_CH_2_)_6_pO(CH_2_)_6_-; Sulfo-Cy5- –Sulfo-Cyanine 5 fluorophore. The structures of 5′- and 3′-modifying groups are given in Figure 2.

## Abbreviations

AMA	a mixture of ammonium hydroxide and 40% aqueous methylamine
BINP	Budker Institute of Nuclear Physics
BNCT	Boron Neutron Capture Therapy
BPA	Boronophenylalanine
BSH	Sodium borocaptate
CI	Cell Index
DAPI	4′,6-Diamidino-2-phenylindole
DIPEA	N,N-Diisopropylethylamine
DMSO	Dimethyl sulfoxide
DSC	N,N′-Disuccinimidyl carbonate
ESI	Electrospray Ionization
IMDM	Iscove’s Modified Dulbecco’s Medium
αMEM	Modified Minimal Essential Medium
MTT	3-(4,5-Dimethylthiazol-2-yl)-2,5-diphenyl tetrazolium bromide
TBDMS	Tert-butyldimethylsilyl
TEA	Triethylamine
THF	Tetrahydrofuran
NHS	N-Hydroxysuccinimide
NMP	N-Methyl pyrrolidone‘
PBS	Phosphate Buffered Saline
RTCA	Real-Time Cell Analysis
TBTA	Tris(benzyltriazolylmethyl)amine
